# Longitudinal Measurements of Tarnished Plant Bug (Hemiptera: Miridae) Susceptibility to Insecticides in Arkansas, Louisiana, and Mississippi: Associations with Insecticide Use and Insect Control Recommendations

**DOI:** 10.3390/insects8040109

**Published:** 2017-10-13

**Authors:** Katherine A. Parys, Randall G. Luttrell, Gordon L. Snodgrass, Maribel Portilla, Josh T. Copes

**Affiliations:** 1 USDA ARS, Southern Insect Management Research Unit, P.O. Box 346, 141 Experiment Station Rd, Stoneville, MS 38776, USA; rglutt@gmail.com (R.G.L.); glsnod@gmail.com (G.L.S.); maribel.portilla@ars.usda.gov (M.P.); 2 Louisiana State University, LSU AgCenter, Northeast Research Station, 4589 Hwy 605, St Joseph, LA 71366, USA; jcopes@agcenter.lsu.edu

**Keywords:** *Lygus lineolaris*, insecticide resistance, bioassays

## Abstract

Concentration-response assays were conducted from 2008 through 2015 to measure the susceptibility of field populations of *Lygus lineolaris* (Palisot de Beauvois) from the Delta regions of Arkansas, Louisiana, and Mississippi to acephate, imidacloprid, thiamethoxam, permethrin, and sulfoxaflor. A total of 229 field populations were examined for susceptibility to acephate, 145 for susceptibility to imidacloprid, and 208 for susceptibility to thiamethoxam. Permethrin assays were conducted in 2014 and 2015 to measure levels of pyrethroid resistance in 44 field populations, and sulfoxaflor assays were conducted against 24 field populations in 2015. Resistance to acephate and permethrin is as high or higher than that previously reported, although some populations, especially those exposed to permethrin, appear to be susceptible. Variable assay responses were measured for populations exposed to imidacloprid and thiamethoxam. Average response metrics suggest that populations are generally susceptible to the neonicotinoids, but a few populations from cotton fields experiencing control problems exhibited elevated LC_50_s. Efforts to associate variability in LC_50_s with recorded use of insecticides and estimated cotton insect losses and control costs suggest that intensive use of insecticides over several decades may have elevated general detoxifying enzymes in *L. lineolaris* and some field populations may be exhibiting resistance to multiple classes of insecticide. These results suggest that efforts should be made to manage these pests more efficiently with a reduced use of insecticides and alternative controls.

## 1. Introduction

Increased use of insecticide sprays for the targeted control of tarnished plant bug (*Lygus lineolaris* (Palisot de Beauvois)) in the Midsouth has been widely discussed and highlighted by numerous authors over the last two decades [1,2]. The current number of sprays made for tarnished plant bug is increasing and is somewhat reminiscent of scheduled “calendar day” approaches to pest management from the 1950s and 1960s that led to adverse effects of over-reliance on chemical control, resulting environmental problems, outbreaks of secondary pests, and a pesticide treadmill that evolved around cycles of new insecticide chemistry [3]. While current insecticides are generally considered to be less harmful to the environment, heavy use of insecticides for plant bugs greatly diminishes the potential economic and environmental advantages of the selective action of transgenic Bt cottons for lepidopteran pests and the successful removal of boll weevil (*Anthonomus grandis* Boheman) as a key pest of southern cotton [4]. *Lygus* spp. have long been a pest of cotton [5], and efforts to manage the pest are critical to profitable cotton production.

Scott and Snodgrass [6], Snodgrass [7], and Luttrell and King [8] provide historical overviews of USDA research and efforts to study the chemical control of tarnished plant bug in the Mississippi Delta over the last 30 years. While the chemical control of tarnished plant bug continues to be a challenge and an economic and ecological problem [9], significant research has been devoted to the topic by federal and state researchers (multiple review articles in Nordlund and Hardee [10] and Edwards [11]). Chemical control options have expanded over the last 30 years, and extension/research entomologists have made efforts to confirm and revise thresholds [12,13]. Researchers at the USDA ARS’ Southern Insect Management Research Unit have produced a wealth of information on tarnished plant bug response to insecticides in laboratory assays [14,15,16,17,18,19,20,21,22], and examinations of resistance mechanisms associated with the variable response of tarnished plant bugs to insecticides [23,24,25,26,27,28]. Research at both the University of Arkansas [29] and Mississippi State University [1] has also addressed similar research topics. Notable published research on the field control of tarnished plant bugs with foliar applied insecticides include Scott et al. [30] and Reed, Jackson, and Harris [2].

Fleming et al. [1] indicated that insecticide use for *L. lineolaris* in the Delta region of Mississippi has increased at a rate of ~0.2 applications per year since 1999, reaching an average of five or more applications per year since 2008. Snodgrass et al. [20,21,31] provided assessments of tarnished plant bug resistance levels to acephate, permethrin, imidacloprid, and thiamethoxam up to 2007. This paper reports the results of continued assays and follow-up information for 2008 through 2015, and possible linkages are explored between measured levels of susceptibility, recorded insecticide use, estimated crop loss, and control costs for the eight-year period.

## 2. Material and Methods

**Figure 1:**
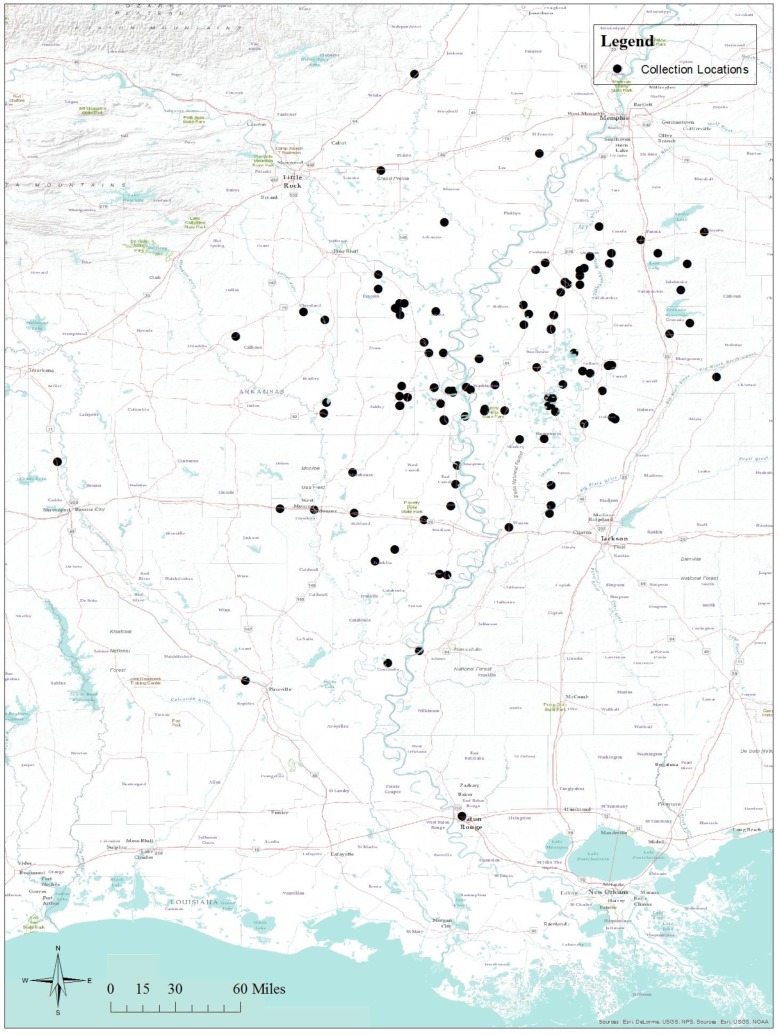
Geographic sites of tarnished plant bug collections in Arkansas, Louisiana, and Mississippi for insecticide assays, 2008–2015.

The methods for the bioassays conducted from 2008 through 2013 were those previously described by Snodgrass et al. [21] for imidacloprid and thiamethoxam assays, and Snodgrass et al. [21] for acephate and permethrin assays. The collection sites (Figure 1) included much of the Delta regions of Arkansas, Louisiana, and Mississippi. Tarnished plant bugs were captured via sweep net primarily from weedy hosts in ditches and field borders as described by Snodgrass et al. [21]. As with previous studies, no attempts were made to associate assay response to host plants due to many collections being made from areas with more than one host present, but the geographic location of the collection site was noted and subsequently studied with specific locations on digital maps. Not all sites had specific geographic coordinates associated with them, and these locations were excluded from some analyses. Depending upon the number of individuals collected at a sample site, assays may have been conducted with multiple insecticides. All of the assays were conducted with adults held under laboratory conditions for 24 h prior to testing; these individuals were held in cardboard (0.95 L) cartons and fed cut-green beans (*Phaseolus vulgaris* L.) surface sterilized by washing in a 3% sodium hypochlorite solution as described by Snodgrass [16].

### 2.1. Glass Vial/Contact Bioassays

Glass vial assays with acephate and permethrin were the same as those described by Snodgrass et al. [21] using the procedures developed by Snodgrass [16]. Technical grade insecticide was purchased from Chem Service (West Chester, PA, USA), stored in a freezer at −20 °C, dissolved in pesticide-grade acetone (Fisher, Fair Lawn, NJ, USA) at known concentrations, and pipetted in 0.5 mL aliquots into 20 mL scintillation vials. Treated vials were rolled on a hotdog roller (Star MFG, Smithville, TN, USA) without heat under a fume-hood until the residues were dry. A 4 cm piece of green bean was added to the vial as a food source before two adult insects were exposed to the insecticide in each vial, with multiple vials making up a replicate. Each population was exposed to five to six concentrations of insecticides, and control vials were treated with acetone alone. Mortality was measured after 24 h of exposure using the criteria outlined in Snodgrass et al. [21]. Assays conducted from 2008 through 2013 were replicated three times, with ten bugs per concentration and replicate. During this period of time, researchers would sometimes return to a sample site to collect additional bugs within 24 h if needed to complete replicates and minimize variability within the collected population. Mortality from bioassays was corrected for control effects using Abbot’s formula [32]. Data were analyzed by probit analysis (PROC PROBIT, SAS 9.4, SAS Institute Inc., Cary, NC, USA, 2013) to establish mortality regression lines, and comparisons were made to those obtained for a control colony from Crossett, AR, considered to be susceptible due to its distal location from insecticide treatments on cotton [20,21,31]. Insects from this location were collected yearly to add to the colony, and reared on broccoli. This colony has been the standard benchmark of susceptibility used by the Snodgrass laboratory for the past two decades.

### 2.2. Floral Foam/Feeding Bioassays

Oral bioassays with imidacloprid and thiamethoxam were developed following observations from Teague and Tugwell [33] that plant bugs feed on fluids in floral foam [20]. The assays conducted between 2008 and 2013 were conducted as described by Snodgrass et al. [20]. Briefly, 12 mm diameter pieces of floral foam were cut from large blocks of floral foam and placed in 20 mL glass scintillation vials. Test concentrations of imidacloprid (0.0, 1.0, 2.5, 5.0, 7.5, and 10 μg/vial) and thiamethoxam (0.0, 0.1, 0.5, 1.0, 2.5, and 5.0 μg/vial) [20] were pipetted onto the floral foam in a 10% honey solution in 0.5 mL aliquots. Observations of mortality for imidacloprid were made at 72 h post-exposure to the treated floral foam, which was based on preliminary observations that indicated low mortality at earlier observation times [20]. The mortality of individuals exposed to thiamethoxam was measured after 24 h of exposure. All of the other insect collection and assay procedures were the same as those previously outlined, including data analyses and replication.

### 2.3. 2014 and 2015 Bioassays

The methodology used in 2014 and 2015 differed slightly, but overall, the procedures were similar to those used by Snodgrass from 2008 through 2013. The differences in the methodology used are noted below. Glass vial assays were essentially the same as those used previously, but the numbers of individuals assayed from each collection were more variable, with a greater emphasis on increasing the number of collections and individual assays. When sufficient numbers of tarnished plant bugs were obtained from a collection, bioassays with six test concentrations were repeated three to four times per field-collected population as described by Snodgrass et al. [21]. The test concentrations used for imidacloprid, thiamethoxam, and sulfoxaflor were 0, 0.15, 0.5, 1.5, 5, and 15 μg/vial, while assays with acephate and permethrin utilized test concentrations of 0, 1, 3, 10, 30, and 100 μg/vial. When the numbers of individuals from a collection were limited, assays were conducted with a minimum of 60 individuals (using six concentrations and 10 bugs per concentration). There were no attempts to return to a collection site in 2014 or 2015 to supplement collection numbers. Data analyses were exactly the same as those previously described, and the resulting data summaries were based only on statistically significant regression models (*p* < 0.05 for Chi-Square Tests of slope and *p* >0.05 for Chi-Square Goodness of Fit). Individual regressions that were not significant with the above criteria were eliminated from both the summary of the Snodgrass laboratory assays for 2008 through 2013 and more recent assays in 2014 and 2015.

The floral foam assays with imidacloprid and thiamethoxam in 2014 and 2015 were exactly the same as those described for the Snodgrass laboratory during 2008 through 2013, except that the observations of mortality for imidacloprid were made at 24 h instead of 72 h based on a series of laboratory assays. Collections made from the Crossett, AR, location were also maintained as a “susceptible” control, collected, and assayed in order to calculate resistance ratios (RR_50_) as a comparison with other locations.

Additionally, in 2014 and 2015, a laboratory colony was added to the routine assays as an experimental control. The laboratory-reared insects were from a colony established in 1998 and kept at the USDA ARS’ Southern Insect Management Research Unit in Stoneville, MS. The colony is reared following details outlined in Portilla et al. [34], to provide large numbers of known-age insects. The insects are reared under controlled conditions in environmental chambers (constant 27 °C and a photoperiod of 16:8 (L:D) h). Assays with the USDA colony were conducted exactly as those with the field colonies, with insects of a uniform age (1-day-old adults) that had been fed on a meridic diet. Groups of mixed-age adults were not available from the USDA colony due to rearing procedures.

Assays with sulfoxaflor have not been previously published, so exploratory assays were conducted using both glass vial and floral foam assays with the USDA laboratory colony. The resulting LC_50_s (95% confidence limits) for the vial assays (9.420 (3.525–27.184) μg/vial) and the floral foam assays (53.236 (12.147–130.639) μg/vial) suggested higher contact activity using the glass vial bioassay. The glass vial procedures as described above were used to test field populations for response to sulfoxaflor in 2015. As with all of the other 2014 and 2015 assays, mortality was measured after 24 h of exposure to the insecticides.

### 2.4. Data Analyses

**Figure 2:**
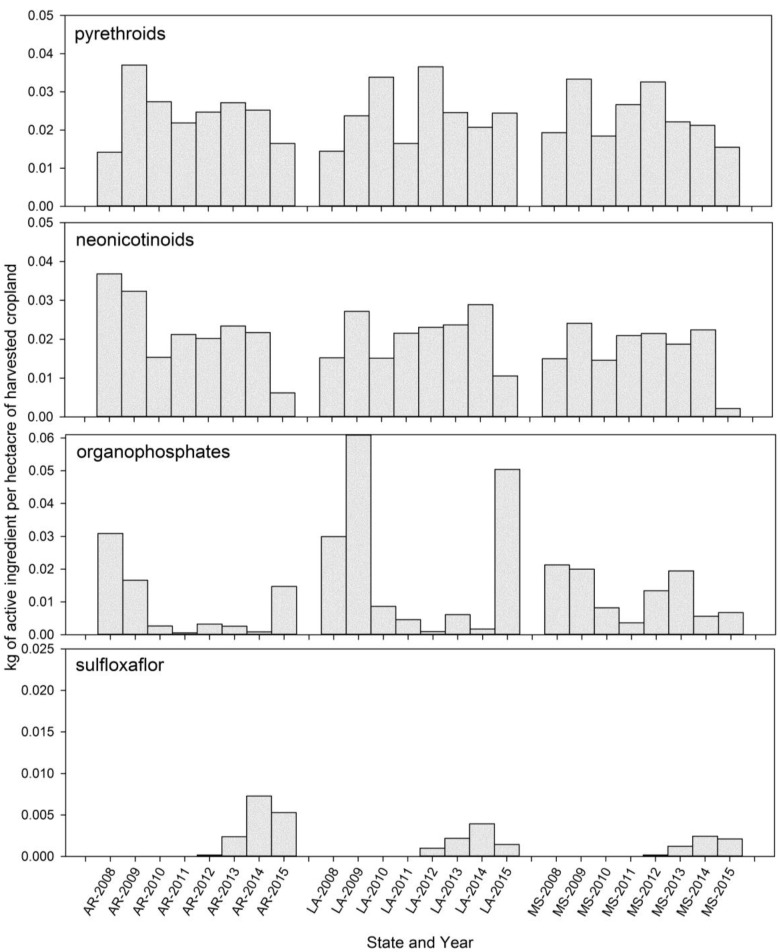
Estimated kg of organophosphate, pyrethroid, neonicotinoid, and sulfoxaflor insecticides applied to cropland in Arkansas (AR), Louisiana (LA), and Mississippi (MS), 2008–2015 [36,37,38].

Once response regressions were completed and the entire assay data set was assembled and available for collective study, we examined the temporal (year) and spatial (state) patterns of response to each insecticide using standard least square ANOVA models in JMP, Version 11.1. The spatial patterns (latitude and longitude) of insect response for each field collection to each insecticide (LC_50_s) were examined by linear models using JMP, Version 11.1. To explore the possible linkages across the landscape for tarnished plant bug susceptibility (average LC_50_s), insecticide use measured as kg of insecticide per hectare of harvested cropland (Figure 2), and annual estimates of cotton insect control costs and losses [35], were averaged across years for each of the three states and studied by Pearson’s pairwise correlation using the Multivariate Procedure in JMP (Version 11.1). Additional exploratory research was conducted by examining possible relationships to estimated insecticide use by collecting county-level information from the USGS National Water Quality Assessment Program’s Pesticide National Synthesis Project [36,37,38] Linear relationships between observed LC_50_s and county-level estimates of kg of insecticide used per hectare of harvested cropland were developed and studied for each of the insecticide classes and the total set of LC_50_ estimates for each insecticide. The insecticide use groups were organophosphates, pyrethroids, neonicotinoids, and sulfoxaflor insecticides. General changes in tarnished plant bug susceptibility over the last two decades were also examined via pairwise correlation analyses relative to recommended control practices by the Mississippi Cooperative Extension Service [39,40,41,42] and annual insect loss estimates for Arkansas, Louisiana, and Mississippi from 2008 through 2015 [35].

## 3. Results

Benchmark comparisons are important criteria in tracking temporal changes in insecticide susceptibility. For tarnished plant bugs in the Midsouth, most of the historical benchmarks are previous studies by the Snodgrass group that utilized field collections from Crossett, AR, as a source of benchmark susceptibility. Table 1 reports recent measurements of insecticide susceptibilities of tarnished plant bugs from the Crossett location.

**Figure 3:**
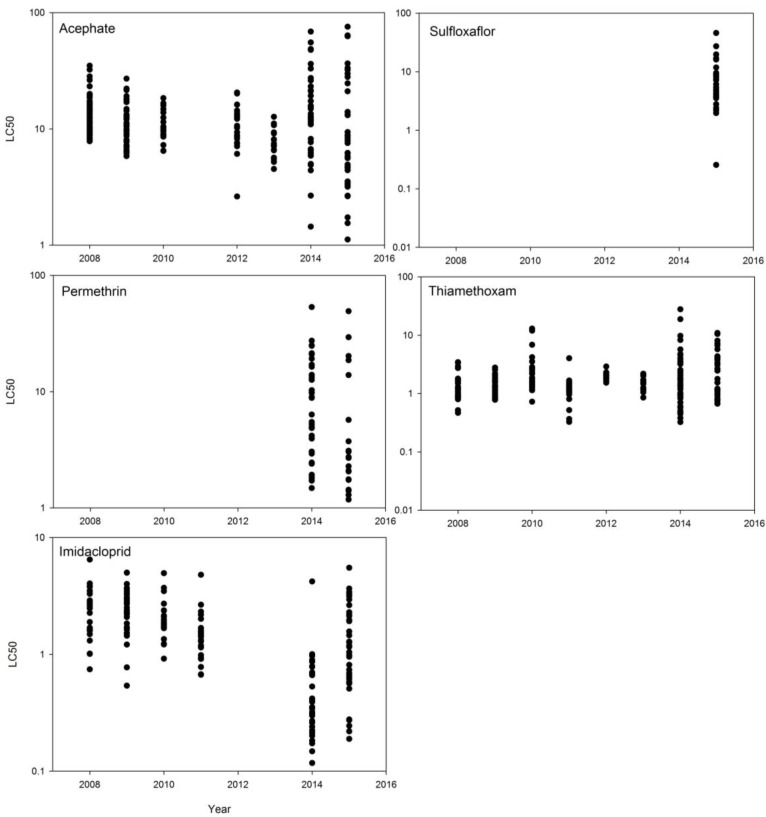
LC_50_s (μg of insecticide/vial) for tarnished plant bug populations from the Mississippi Delta exposed to acephate (vial 24 h), sulfloxaflor (vial 24 h), permethrin (vial 24 h), thiamethoxam (foam 24 h), and imidacloprid (foam 72 h 2008–2013; foam 24 h 2014–2015); LC_50_s from 2014–2015 are converted (by 3.33) to be on the same relative scale as previous bioassays from 2008 to 2015.

The annual variability among field populations of tarnished plant bug in measured LC_50_s for acephate, imidacloprid, thiamethoxam, permethrin, and sulfoxaflor is illustrated in Figure 3. Table 2 summarizes the overall assay data for 2008–2015 with LC_50_s averaged across all significant regressions, including all significant assays with field populations and all significant assays conducted using the USDA lab colony. The USDA lab colony has only once previously been reported as an experimental control [14]. Table 3, Table 4, Table 5 and Table 6 compare annual measurements of susceptibility to previous Snodgrass benchmarks [20] and assays with the more recent assays with insects from the Crossett control location (Table 1).

### 3.1. Glass Vial/Contact Bioassays

#### 3.1.1. Acephate Assays

A total of 252 regressions described individual field and laboratory assays with acephate. Of these, 229 (91%) were measurements of different field collections with an average LC_50_ of 12.249 μg/vial (Table 2). The average LC_50_ for the USDA laboratory colony (25.509 μg/vial) was significantly higher than the average LC_50_ for field collections based on a non-overlap of 95% fiducial limits (FL). The laboratory colony also exhibited wide variability in response to acephate (12-fold differences in LC_50_s among the 23 individual tests). Field populations varied more than 60-fold with a maximum LC_50_ of 68.669 μg/vial. The highest average LC_50_ was observed in 2006 (16.1 μg/vial). No assays were conducted with acephate in 2011 (Table 3 and Figure 2). When the collective acephate data were studied by analysis of variance (ANOVA), no significant difference was found in average LC_50_s among collections from the three different states (df = 2, F = 1.7919, *p* = 0.1691), though differences among years (df = 6, F = 4.0498, *p* = 0.0007) were detected. The highest annual average LC_50_ was observed in 2014 (19.079 μg/vial) (Table 3). LC_50_ was negatively associated with latitude (n = 165, r^2^ = 0.0259, Intercept ± standard error (SE) = 69.3435 ± 28.0621, Slope ± SE = −1.7512 ± 0.8407) but not longitude (n = 165, r^2^ = 0.0075, F = 1.2348, *p* = 0.2681). Most of the populations had LC_50_s with a lower fiducial limit greater than 3.6 (the upper level fiducial limit (reported by Snodgrass as confidence limit) reported for the Crossett location [21]). From 4% (2015) to 52% (2008) of the average annual LC_50_s observed were significantly more than those at the Crossett location in 2014 (Table 3).

#### 3.1.2. Permethrin Assays

Wide variability was observed in the responses of field collections (n = 44) and the lab colony (n = 26) to permethrin assays in 2014 and 2015 (Table 2). We observed a similar range of responses to those previously reported by Snodgrass et al. [31] across the 44 field colonies tested in 2014 and 2015 (0.284–53.414 μg/vial), though a few populations were more susceptible (Figure 2).

As with other assays conducted, there were no differences in LC_50_s among states (df = 2, F = 0.6358, *p* = 0.5349). The average LC_50_ for 2014 (9.828 μg/via) was statistically higher than the average for 2015 (5.722 μg/vial) (df = 1, F = 5.5246, *p* = 0.0239), but this may not be important given the wide variability among samples for both years (Figure 3). There was a significant negative effect of latitude (n = 52, F = 4.3039, *p* = 0.0431) of collection site on permethrin LC_50_s in 2014 and 2015 (r^2^ = 0.0778, Intercept ± SE = 116.75173 ± 53.8576; Slope ± SE = −3.351498 ± 1.615499)). There was no effect of longitude (n = 52, F = 0.0010, *p* = 0.9745).

Using comparisons to former Snodgrass data (Table 4), none of the populations tested had 95% fiducial limits greater than the highest upper fiducial limit (reported by Snodgrass as confidence limit) reported for a field population (77.4 μg/vial) [31]. Three (7%) had LC_50_s with lower fiducial limits greater than the upper fiducial limit reported for the Crossett location, while 13 of the 45 (33%) had fiducial limits that did not overlap with the limits estimated from the Crossett location in 2014 (Table 1). One population of interest was a field population from cotton in Humphreys County, MS, that experienced control problems with thiamethoxam. The measured LC_50_ for this population was 5.493 (2.080–16.226) μg/vial, high enough to be included in the group of colonies with fiducial limits greater than the Crossett control. A corresponding regression model with thiamethoxam for this collection location was eliminated from the overall summary because the model was not statistically significant.

### 3.2. Floral Foam/Feeding Bioassays

#### 3.2.1. Imidacloprid Assays

Imidacloprid assays for 2008–2013 measured mortality at 72 h post-exposure to insecticide treatment. The average LC_50_ for the field populations was 2.209 μg/vial with a 12-fold range in response, while the maximum LC_50_ was 6.470 μg/vial (Table 2). Assays in 2014 and 2015 measured mortality at 24 h. Of the 51 field collections tested and 22 assays with the USDA lab colony, an average LC_50_ of 3.352 μg/vial was observed. Differences between the field collections and the USDA lab colony were not observed, but high variability was observed among the field populations (36-fold). Regression models were developed from comparative assays and observed varying times using floral foam assays for tarnished plant bug mortality; at 24 h (n = 400, slope ± SE = 1.1422 ± 0.1222, Chi-Square 87.62 (*p* < 0.0001), LC_50_ (95% FL) of 14.084 (10.597–19.779)), 48 h (n = 400, slope ± SE = 1.3457 ± 0.1275, Chi-Squre 111.41 (*p* < 0.0001), LC_50_ (95% FL) of 11.1916 (8.8788–14.653)), and 72 h (n = 400, slope ± SE = 1.4641 (0.1349), Chi-Square 117.90 (*p* < 0.0001), LC_50_ (95% FL) of 4.219 (3.3542–5.267)). These suggested a 3.33-fold difference in LC_50_s measured at 24 h and 72 h for the USDA laboratory colony exposed to imidacloprid in the floral foam assays. The mortality was lower at 24 h as indicated by Snodgrass et al. [20], and the efficiency of making all observations on the same day facilitated the ability to conduct additional assays. While the change in methodology between research programs was not ideal, it may actually underestimate susceptibility. If assay data observed in 2014 and 2015 are divided by 3.33 to correct for differences in observation time as described in the methods section, the populations assayed in 2014 and 2015 were at least as susceptible as those assayed from 2008 through 2013.

Imidacloprid LC_50_s were significantly influenced by year of assay (df = 5, F = 6.1881, *p* < 0.0001). The average LC_50_ for the 24 field populations assayed in 2015 (5.029 μg/vial) was greater than the average of the populations assayed in other years (Table 5). There were no observed differences among states (df = 2, F = 0.1981, *p* = 0.8205), and LC_50_ values were not influenced by the latitude (n = 146, F = 0.7297, *p* = 0.3944) or longitude (n = 146, F = 2.5675, *p* = 0.1113) of the collection location.

Comparisons of LC_50_s to those previously reported by Snodgrass [20] and the 2008 measurement of imidacloprid susceptibility in the Crossett control generally found no evidence of a changed susceptibility to imidacloprid (Table 5). One collection made in 2008 from Concordia Parish, LA, had an elevated response (LC_50_ (95% FL) of 6.470 (4.351–12.761)). In the 2014 and 2015 assays, a colony collected from a cotton field experiencing control problems in Tallahatchie County, MS, had an LC_50_ of 13.997 μg/vial when measured at 24 h, and the estimated LC_50_ at 72 h would be 4.203 μg/vial. Many of the field populations tested had LC_50_s with lower fiducial limits greater than 1.09 μg/vial (the upper fiducial limit (reported by Snodgrass as confidence limit) reported for the Crossett control [20]). Half of the collections tested in 2009 and 2010 had LC_50_s greater than the Crossett control population.

#### 3.2.2. Thiamethoxam Assays

A total of 208 field populations were studied for susceptibility to thiamethoxam over an eight-year period (Table 2). The average LC_50_ was 2.066 μg/vial, with two populations in 2010 and two populations in 2014 exhibiting LC_50_s in excess of 10 μg/vial (Figure 2). Two of the collections were made in August of 2014 (with a maximum LC_50_ measured of 27.213 μg/vial) from the same cotton field that was experiencing control problems, taken approximately a week apart in Tallahatchie County, MS. The insects from this collection exhibited high LC_50_s to thiamethoxam, acephate, and permethrin. Thiamethoxam assays with the USDA lab colony (n = 16) produced LC_50_s slightly higher than those reported by Snodgrass et al. [20] for the field colonies, but not significantly different than the Crossett location (Table 1).

Similar to the imidacloprid assays, no differences were observed in LC_50_s for thiamethoxam among states (df = 2, F = 0.1581, *p* = 0.8539), but there was a significant effect of year (df = 7, F = 2.1952, F = 0.0363). However, Tukey’s HSD test failed to separate differences among years at *p* = 0.05. The highest average LC_50_ was recorded for 2014 (3.375 μg/vial), while the lowest was in 2011 (1.255 μg/vial) across the 25 populations tested (Table 6). No significant influences of latitude (n = 201, F = 0.0022, *p* = 0.9623) or longitude (n = 201, F = 0.7254, *p* = 0.3954) associated with collection site on the resulting LC_50_s were detected.

Five collections from 2014 and three collections from 2015 had average LC_50_s greater than 4.16 μg/vial. The upper level fiducial limit for the Crossett control colony was 3.563 μg/vial in 2010 (Table 6). Two of the 27 populations tested in 2010 had LC_50_s with lower fiducial limits greater than 3.563 μg/vial; both of these were populations collected about a week apart during June from two differing locations in Humphreys County, MS. Three of the 32 populations tested in 2014 had LC_50_s with lower fiducial limits greater than 3.564 μg/vial. None of the populations tested were significantly different than the Crossett control colony in 2014 (3.298 μg/vial) (Table 1).

#### 3.2.3. Sulfoxaflor Assays

Benchmark data for sulfoxaflor were not previously obtained because of the unavailability of technical grade insecticides prior to the commercial use of the formulated product. Data collected in 2015 were taken several years after the initial use of the insecticide, and field populations may have already experienced some selection. For the 21 field populations tested in 2015 for susceptibility to sulfoxaflor using a glass vial bioassay and 24 h observations, an average LC_50_ of 9.042 μg/vial was measured with 95% confidence limits of 4.924 to 13.159 μg/vial. The range in response across the field populations was high (179-fold) as compared to the range in response for the USDA Laboratory colony (11-fold). Average LC_50_s were similar for the field and laboratory assays (Table 2).

### 3.3. Relationships to Insecticide Use and Control Recommendations

Estimated insecticide use by class of insecticide was obtained for all counties/parishes in Arkansas, Louisiana, and Mississippi for the years corresponding to our bioassays from the USGS National Water Quality Assessment Program, Pesticide National Synthesis Project. The average annual kg of insecticide per hectare of harvested crop land are summarized and plotted in Figure 2 to illustrate the overall annual use patterns for organophosphates, pyrethroids, neonicotinoids, and sulfoxaflor insecticides among the different states. Pyrethroid use was not significantly different among states (df = 2, F = 0,0471, *p* = 0.9540), and the interaction between states and years was not significant (df = 14, F = 1.1823, *p* = 0.2823). Pyrethroid use was significantly different across years (df = 7, F = 3.5852, *p* = 0.0008). More pyrethroid insecticide was used in 2009 than in 2015 and 2008 (Figure 2). Organophosphate use was significantly influenced by an interaction between states and years (df = 14, F = 5.5.9524, *p* < 0.0001). The use of organophosphates in Louisiana during 2009 and 2015, and in Arkansas during 2008, was greater than the amount used in Arkansas in 2010, 2011, and 2014 and that used in Louisiana in 2012 and 2014. Neonicotinoid insecticide use was also influenced by year (df = 7, F = 9.9814, *p* < 0.0001), but not by state (df = 2, F = 1.4401, *p* = 0.2372) or the interaction between year and state (df = 14, F = 0.3760, *p* = 0.9816). The largest amount of neonicotinoids per hectare of harvested cropland was in 2009 and 2014, while the least was in 2008 and 2015. For the four years of measured sulfoxaflor use, there were significant effects of state (df = 2, F = 9.3893, *p* = 0.0001) and year (df = 3, F = 15.2742, *p* < 0.0001), but the interaction of state and year (df = 6, F = 2.0076, *p* = 0.0638) was not significant. A greater amount of sulfoxaflor was applied in Arkansas than in Mississippi, and the amount of chemical used per acre of harvested cropland in Louisiana did not differ from that of the other two states. Overall, more sulfoxaflor was used in 2014 and 2015 than in 2012 and 2013. Table 7 provides regression models that illustrate the connections among insecticide use across the different insecticide classes.

Subsets of these data associated with individual counties and parishes for our sample sites (Figure 1) were created and paired with our measured LC_50_s to study possible relationships between insecticide use and resulting LC_50_s. LC_50_s for the different insecticides were also examined for paired linear relationships. The resulting regression models are reported in Table 8. Acephate LC_50_s were positively related to permethrin LC_50_s (n = 40, r^2^ = 0.1554, F = 8.0958, *p* = 0.0067) and sulfoxaflor LC_50_s (n = 20, r^2^ = 0.2984, F = 4.919, *p* = 0.0127) in 2014 and 2015. There were no other significant predictors of acephate LC_50_ among the insecticide use and insecticide assay data sets. Permethrin LC_50_s were marginally related to kg of pyrethroids applied per hectare of harvested cropland (n = 43, r^2^ = 0.0641, F = 2.8699, *p* = 0.1015) and kg of neonicotinoids applied per hectare of harvested cropland (n = 43, r^2^ = 0.0804, F = 3.5827, *p* = 0.0655). Imidacloprid LC_50_s were negatively related to kg of neonicotinoids per hectare of harvested cropland (n = 146, r^2^ = 0.0367, F = 5.4920, *p* = 0.0205). LC_50_s for imidacloprid were related to LC_50_s for permethrin (n = 48, r^2^ = 0.1608, F = 8.8169, *p* = 0.0047), sulfoxaflor (n = 24, r^2^ = 0.1682, F = 4.4494, *p* = 0.0465), and highly related to LC_50_s for thiamethoxam (n = 136, r^2^ = 0.1794, F = 30.1680, *p* < 0.0001). As with the imidacloprid LC_50_ comparisons, LC_50_s for thiamethoxam were highly related to those for imidacloprid (Table 8). There were no other significant relationships between thiamethoxam LC_50_s or sulfoxaflor LC_50_s with insecticide use and the insecticide assay data.

The comparisons were further refined by calculating average LC_50_s for each county/parish to compare to the annual average insecticide use data for counties/parishes (Table 9). Significant (*p* < 0.05) regression models included positive relationships between kg of pyrethroids and kg of sulfoxaflor applied per hectare of cropland and resulting LC_50_s for permethrin (Table 9). Thiamethoxam LC_50_s were a positive predictor of imidacloprid LC_50_s, while the amount of pyrethroid insecticide applied per acre of harvested cropland was a negative predictor of imidacloprid LC_50_ (Table 9).

Recommendations for the control of tarnished plant bug in cotton by the Mississippi Cooperative Extension Service are summarized in Table 10 for the years 1983, 1993, 2003, and 2013. These snapshots of time are presented to illustrate the evolution of plant bug control procedures and changing availability of and preferences for different insecticides. The recommended insecticides in 1983 and 1993 were almost entirely organophosphates, with the exception of the carbamate carbaryl in 1983 and oxamyl in 1993. The four organophosphates still recommended in 2013 were all recommended at use rates generally two- to three-fold higher than those recommended in 1983, with the exception of malathion, which was still recommended at a similar rates (Table 10). Three classes of insecticide chemistry were available for use in 2003, and six different types of insecticide chemistries were available for plant bug control in 2013.

Annual insect loss estimates included the number of insecticide applications for plant bugs, number of insecticide applications for bollworms, acres of cotton harvested, yield, percent crop loss to insects, average number of total insecticides sprays per year, and cost of all foliar insecticides. Significant correlations were observed between a variety of variables examined (Table 11). No significant correlations (*p* = 0.05) were observed for estimated percent crop loss to insects, average LC_50_s for sulfoxaflor, average LC_50_s for imidacloprid, or kg of sulfoxaflor applied per acre of harvested cropland.

## 4. Discussion

This paper continues a history of the reporting of assay responses of tarnished plant bug to the major classes of insecticide used in the Delta. There is a wealth of previous information on tarnished plant bug response to insecticides in this area, including early work [43,44], numerous papers from the Snodgrass laboratory [7,14,15,16,17,19,20,21,22,45], studies in Arkansas [29,33], studies in Louisiana [46,47], a summary of small-plot field experiments in Mississippi [2], and a number of efforts to understand tarnished plant bug resistance mechanisms [1,23,25,26,27,28]. Understanding the changes in susceptibility over time are important, but understanding how these changes evolve and how management practices can be refined with this information is particularly relevant to managing tarnished plant bugs in the future. Snodgrass [7] concluded a summary of 30 years of research with the tarnished plant bug in the Mississippi Delta by writing “as long as insecticides are the main method for controlling TPB (tarnished plant bug) in cotton, the TPB will remain a serious pest”.

While the data presented here include previously unpublished resistance information from the Snodgrass laboratory, it also attempts to transition the research to new approaches. Research conducted in 2014 and 2015 introduced the use of the USDA lab colony as an experimental control. The historical use of collections from Crossett, AR, as an index of susceptibility may not be sustainable (Table 1), and the historical approach potentially confounds insect nutrition and age with measurements of insecticide susceptibility. Indirect comparisons back to previous published benchmarks are useful and important, but paired comparisons to a susceptible lab colony would strengthen experimental measurements and allow researchers to control experimental error. Both Zhu and Luttrell [23] and Zhu et al. [24] made comparisons between field-collected strains and a meridic diet-fed laboratory colony of tarnished plant bugs, and Fleming et al. [1] discussed their omission of a laboratory-susceptible strain and the possibility that all of their strains may have had some level of insecticide resistance. The high tolerance and variability of the USDA lab colony to acephate, permethrin, and thiamethoxam needs additional research if it is to be used as an experimental control. Regardless, there needs to be more experimental consistency and more understanding of the relationships between laboratory susceptibility and control in the field. Because of the high tolerance and variability observed with the lab colony, comparisons of insecticide susceptibility were based on previously published benchmarks from the Snodgrass laboratory using insects from Crossett and our more recent direct measurements of response from field collections of insects from the Crossett location (Table 1). Based on these traditional benchmark comparisons to the Crossett susceptible location, the USDA lab colony would be judged to be resistant to most of the insecticides tested. The variability of response in the USDA lab colony was greatest for acephate and permethrin assays, while the variability with the neonicotinoids and sulfoxaflor was less, perhaps suggesting the presence of resistance genes for acephate and pyrethroid resistance within the USDA lab colony. A more plausible explanation is likely linked to the differences in food between the lab colony’s meridic diet and the field-collected insects that have fed on a wide range of nutritionally variable native plants and crops. Nutrition and host plant development have been shown to affect measurements of insecticide susceptibility in several other insect species [48,49,50,51,52].

Based on the acephate assay results of this study (Table 2 and Table 3, Figure 2) and comparisons to declining levels of susceptibility reported by Snodgrass et al. [21], tarnished plant bug populations in the Delta region of Arkansas, Louisiana, and Mississippi have high frequencies of resistance to this organophosphate insecticide. However, there is still wide variability among populations and some populations appear to be relatively susceptible (Figure 2). Research by others [1,23,24] has found similar results and elevated activities of metabolic enzymes in field populations collected from the Delta region. Both Zhu and Luttrell [23] and Fleming et al. [1] have reported elevated levels of esterase activity in Delta populations of tarnished plant bugs. Zhu and Luttrell [23] associated these elevated levels with reduced susceptibility to acephate, while Fleming et al. [1] observed differences in esterase activities in bugs from two different regions of Mississippi, but did not associate the differences with individual assay results. Both Zhu and Luttrell [23] and Fleming et al. [1] measured variable levels of glutathione S-transferase in the Delta populations of tarnished plant bug. Zhu and Luttrell [23] indicated that inhibitors of glutathione S-transferase exhibited less suppression in 2010 as compared to 2006, and suggested that this may indicate a potential shift in the genetics of the pest populations. Recommended application rates of acephate were increased from 0.23–0.33 lb ai/acre to 0.5–1.0 lb ai/acre during this time period (Table 10), indicating a growing concern for the level of field control being achieved. Reed et al. [2] summarized results of replicated field experiments conducted to measure tarnished plant bug control in cotton with organophosphate insecticides, and reported that the average control measured from 1982 to 1997 was 57%. If this level of field control compares to the previous assay data of Snodgrass et al. [21], and field control is even loosely related to changes in assay response, acephate applications alone do not adequately control tarnished plant bugs in the field. Bioassays were conducted with permethrin only in 2014 and 2015, as most of the previous systematic monitoring for pyrethroid resistance was based on the use of a diagnostic-dose assay [18,20]. Based on our 2014 and 2015 assays (Figure 3 and Table 4), tarnished plant bugs still express resistance to permethrin. Pyrethroid-resistant populations are common, but there is wide variability in response among populations and some are relatively susceptible. Perhaps, a return to careful monitoring of populations prior to spraying a pyrethroid would enhance the efficiency of insecticide selection decisions and allow growers to more carefully determine when to use pyrethroid insecticides [18].

Snodgrass et al. [21] reported variability in the response of tarnished plant bugs to both imidacloprid and thiamethoxam, and suggested that some imidacloprid resistance was present. At the time, it was also generally concluded that the neonicotinoids were the only widely used insecticides to which tarnished plant bug populations are still susceptible. In our studies (Table 2 and Table 5, Figure 2), we found no evidence of increasing resistance to imidacloprid, although the populations commonly had higher LC_50_s than the Crossett collections. With thiamethoxam, a few colonies collected from cotton fields experiencing control problems, in particular the colony collected from Tallahatchie County, MS, in 2014, were reason for concern, as was a population with an elevated response in 2010 (Figure 2). Because of the importance of these insecticides to the effective control of tarnished plant bugs, and the possible selection for elevated esterase, P450, and glutathione S-transferase genes that may confer resistance to multiple classes of insecticides [24], new approaches to tarnished plant bug control that lessen the current intensive and repeated use of insecticides of all classes are needed. As with the other insecticide classes, additional research is needed to associate variability in laboratory assays to insect survival and crop damage in the field.

Those bioassays conducted with sulfoxaflor may serve as future benchmarks, but these data were collected several years after the insecticide chemistry was commercially deployed and variability was observed in our 24 h glass vial assays (Figure 3, Table 2). Additional research is needed to refine the assay methods and perhaps extend the observation time, but as with the other insecticides, these studies need to be related to field observations of plant bug survival. The lack of benchmark information pre-commercial release of sulfoxaflor may hinder future resistance assessments.

Estimated insecticide use in Arkansas, Louisiana, and Mississippi varied considerably across years and states depending on the chemical class. Regressions between the estimated use of the four insecticide classes were all highly significant when all three states were pooled. Thiamethoxam LC_50_s were a positive predictor of imidacloprid LC_50_s, while the amount of pyrethroid insecticide applied per acre of harvested cropland was a negative predictor of imidacloprid LC_50_. The subset data utilizing only counties and parishes with collection records revealed linkages between LC50 values and estimated use for several classes of insecticides (Table 8). The observations of linkages between pyrethroids and neonicotinoids in measured LC_50_s and the positive influence of estimated pyrethroid use on neonicotinoid susceptibility is concerning, especially in light of the research of Zhu and Luttrell [23], Zhu et al. [24], and Fleming et al. [1] that report elevated levels of broad-based metabolic enzymes capable of detoxifying multiple classes of insecticides.

Thirty years of tarnished plant bug thresholds and insecticide recommendations from Mississippi State University’s Cooperative Extension Service provide snapshots of the evolution of thresholds, changes in control strategies, and availabilities of different classes of insecticides. Thresholds have been refined [12,13] with adjustments for relative critical densities among different sampling procedures, and treatment levels have generally decreased (i.e., sprays recommended at lower plant bug densities) as the recency of recommendations increased (Table 10). For the first time since pyrethroids were introduced into Mississippi cotton production in 1979 [53], specific pyrethroid insecticides are now listed in combination with other insecticides as recommended options for the control of plant bugs and fleahoppers [54]. Additionally, pyrethroids are also no longer recommended for the control of bollworms and budworms for the first time since 1979. Correlations utilizing data from the annual loss estimates [35] are not unexpected, with significant positive relationships observed between the total numbers of foliar applications, the number of applications made for tarnished plant bugs, and the cost of all foliar applications.

## 5. Conclusions

The exploratory examination of associations among measured plant bug susceptibility, reported insecticide use for individual counties/parishes, and estimated cotton insect losses and control costs is likely influenced by a number of spurious relationships, including temporal and spatially dynamic patterns of cotton acreage across the landscape. Hopefully, the detailed information on tarnished plant bug susceptibility to insecticides in the Delta over time will allow future pest managers to develop more efficient and sustainable insect management programs with lessened use of insecticide. Developing alternative control options should be a priority for future research [55]. Given the wealth of historical assay data on tarnished plant bug susceptibility to insecticides and the ability to link this historical information to future research, a priority should be placed on field research, especially additional empirically based research comparing varying levels of tarnished plant bug control across the range of insecticide tools available and including assays to link historical data. Empirical, field-based research will enhance our understanding of how the variable expressions of survival in assay experiments relate to the survival of tarnished plant bugs and crop damage in insecticide-treated fields.

## Figures and Tables

**Table 1:** Concentration-response regressions for insects collected as a reference control from Crossett, AR, during 2008–2014.

Year	Insecticide	Significance of Slope			LC_50_ (95% FL) ^a^	Goodness of Fit	
		Slope ± SE	*X*^2^	*p* = *X*^2^		*X*^2^	*p* = *X*^2^
2008	imidacloprid	1.295 ± 0.3754	11.89	0.0006	0.743 (0.0001–4.1288)	12.24	0.0066
2008	thiamethoxam	1.322 ± 0.218	36.76	<0.0001	0.868 (0.5639–1.2867)	1.5	0.6826
2010 ^b^	thiamethoxam	0.739 ± 0.2422	9.30	0.0023	1.741 (0.6343–3.564)	0.09	0.9925
2010 ^b^	thiamethoxam	1.827 ± 0.2828	41.72	<0.0001	1.858 (1.3593–2.4751)	2.99	0.393
2014	acephate	2.043 ± 0.5611	18.58	0.00345	6.54 (2.1902–9.1719)	4.68	0.3701
2014	thiamethoxam	1.521 ± 0.3986	15.06	0.0012	3.298 (1.6882–12.8357)	1.61	0.6601
2014	permethrin	2.727 ± 0.7891	12.42	0.0019	1.8804 (1.1169–3.2204)	5.26	0.4338

^a^ All LC_50_s are expressed as μg/vial. The comparative Crossett control LC_50_s (95% fiducial limit (FL)) for thiamethoxam and imidacloprid are 1.52 (1.00–2.49) μg/vial and 0.85 (0.61–1.09) μg/vial, respectively [31]. The comparative Crossett control LC_50_s for permethrin and acephate are 3.5 (2.7–4.4) μg/vial and 3.1 (2.6–3.6) μg/vial, respectively [21]. ^b^ Two separate collections were made in 2010 (3rd June and 22nd June), and both were screened against thiamethoxam.

**Table 2:** Summary statistics of bioassays (2008–2015) conducted to measure susceptibility of tarnished plant bug populations in the Mississippi Delta to acephate, imidacloprid, permethrin, sulfoxaflor, and thiamethoxam.

	No. Assays	Mean LC_50_^a^	Minimum LC_50_ Observed	Maximum LC_50_ Observed	95% FL (All Assays)
**Acephate Vial 24 h**	252	13.4594	1.1165	75.6289	12.1711–14.7477
Field	229	12.2491	1.1165	68.6697	11.1416–13.3567
Lab	23	25.5089	6.1635	75.6289	18.2292–32.7886
**Imidacloprid Foam 72 h**	95	2.2095	0.5383	6.47017	1.9814–2.4375
Field	95	2.2095	0.5383	6.47017	1.9814–2.4375
Lab	0	-	-	-	-
**Imidacloprid Foam 24 h**	73	3.3524	0.3897	18.3516	2.5355–4.1693
Field	51	3.1004	0.3897	13.9973	2.2129–3.9879
Lab	22	3.9366	0.6273	18.3516	2.1602–5.7130
**Permethrin Vial 24 h**	70	8.0589	0.2837	53.4136	5.5770–10.5408
Field	44	5.3533	0.2837	53.4136	2.7046–8.0020
Lab	26	12.6376	0.8881	49.1872	8.1403–17.1348
**Sulfoxaflor Vial 24 h**	29	8.9041	0.2557	45.8167	5.5352–12.2730
Field	21	9.0421	0.2557	45.8167	4.9245–13.1597
Lab	8	8.5420	2.3993	27.1840	2.4339–14.6500
**Thiamethoxam Foam 24 h**	224	2.2727	0.3230	27.7130	1.9074–2.6381
Field	208	2.0664	0.3230	27.7130	1.7025–2.4302
Lab	16	4.9555	1.1860	10.8960	3.5271–6.3838

^a^ All LC_50_s expressed as μg of insecticide/vial.

**Table 3:** Summary of annual responses of tarnished plant bug field population responses to acephate *.

	No. of Field Populations	Average LC_50_^a^	Minimum LC_50_^a^	Maximum LC_50_^a^	No. LC_50_s ^a^ >16.9 ^b^	No. Lower FL >3.6 ^c^	No. Lower FL >9.2 ^d^
2008	71	12.539	7.823	34.922	11	69	37
2009	41	11.869	5.834	27.051	8	41	13
2010	24	10.624	6.467	18.410	1	24	8
2011	-	-	-	-	-	-	-
2012	21	11.240	2.622	10.510	3	21	8
2013	18	7.864	4.515	12.702	0	18	1
2014	29	19.079	1.440	68.670	3	27	7
2015	21	11.171	1.117	62.653	2	15	1

^a^ All LC_50_s expressed as μg of insecticide/vial. ^b^ 16.9 μg/vial is the highest upper level fiducial limit (reported by Snodgrass as confidence limit) reported for a field population [21]. ^c^ 3.6 μg/vial is the upper level fiducial limit (reported by Snodgrass as confidence limit) reported for the Crossett control population [21]. ^d^ 9.2 μg/vial is the upper level fiducial limit (reported by Snodgrass as confidence limit) measured for the Crossett control population in 2014 (Table 1). * Using Snodgrass’ [21] criteria of a 3-fold resistance ratio (RR_50_) as an indicator of resistance, 59 (82% in 2008), 24 (58% in 2009), 14 (58% in 2010), 13 (62% in 2012), 18 (28% in 2013), 11 (96% in 2014), and 6 (47% in 2015) of the populations tested were resistant and would be difficult to control with acephate in the field. Using the higher LC_50_ measured for the Crossett colony in 2014 (6.54 μg/vial, Table 1), the number of colonies expressing resistance would be 7 (10% in 2008), 3 (7% in 2009), 0 (0% in 2010), 2 (9% in 2012), 0 (0% in 2012), 11 (38% in 2014), and 6 (29% in 2015).

**Table 4:** Summary of annual responses of tarnished plant bug field population responses to permethrin ^a^.

	No. of Field Populations	Average LC_50_^b^	Minimum LC_50_^b^	Maximum LC_50_^b^	No. LC_50_s ^b^ >77.4 ^c^	No. Lower FL >4.4 ^d^	No. Lower FL >1.61 ^e^
2014	29	9.828	0.307	53.414	0	3	13
2015	16	5.722	0.284	49.187	0	0	2

^a^ No data collected from 2008 to 2013. ^b^ All LC_50_s expressed as μg of insecticide/vial. ^c^ The highest upper fiducial limit (reported by Snodgrass as confidence limit) reported for the field population was 77.4 μg/vial [21]. ^d^ The fiducial limit (reported by Snodgrass as confidence limit) [21] for the Crossett control colony is 4.4 μg/vial. ^e^ The upper fiducial limit measured for the Crossett control colony in 2014 was 1.61 μg/vial (Table 1). Two populations in 2014 had LC_50_s >24 μg/vial and would be considered resistant by Snodgrass [31].

**Table 5:** Summary of annual responses of tarnished plant bug field population responses to imidacloprid.

	No. of Field Populations	Average LC_50_^a^	Minimum LC_50_^a^	Maximum LC_50_^a^	No. LC_50_s ^a^ >10.68 ^b^ (35.56 for 2014–15)	No. Lower FL >1.09 ^c^ (3.63 for 2014–15)	No. Lower FL >4.128 ^d^ (13.746 for 2014–15)
2008	23	2.526	0.743	6.470	0	10	1
2009	29	2.518	0.538	5.008	0	19	0
2010	19	2.162	0.916	4.949	0	11	0
2011	25	1.640	0.669	4.800	0	3	0
2012	-	-	-	-	-	-	-
2013	-	-	-	-	-	-	-
2014	28	1.804	0.390	13.997	0	1	0
2015	24	5.029	0.297	18.352	0	4	0

^a^ All LC_50_s expressed as μg of insecticide/vial. ^b^ The highest upper fiducial limit (reported by Snodgrass as confidence limit) is 10.68 μg/vial [20]. The projected estimate for a 24 h response based on a 3.33-fold difference in LC50s for 24 and 72 h is 35.56 μg/vial. ^c^ The upper fiducial limit (reported by Snodgrass as confidence limit) for the Crossett control is 1.09 μg/vial [20], while 3.63 is the projected 24 h upper level fiducial limit. ^d^ The upper fiducial limit measured for the Crossett control population in 2008 (Table 1) is 4.128 μg/vial, while 13.746 is the projected 24 h response.

**Table 6:** Summary of annual responses of tarnished plant bug field population responses to thiamethoxam.

	No. of Field Populations	Average LC_50_^a^	Minimum LC_50_^a^	Maximum LC_50_^a^	No. LC_50_s ^a^ >4.16 ^b^	No. Lower FL >3.564 ^c^	No. Lower FL >13.8955 ^d^
2008	27	1.404	0.467	3.431	0	0	0
2009	25	1.398	0.778	2.771	0	0	0
2010	27	2.810	0.722	12.907	4	2	0
2011	25	1.255	0.325	4.106	0	0	0
2012	21	2.003	1.528	2.889	0	0	0
2013	22	1.509	0.849	2.172	0	0	0
2014	32	3.375	0.323	27.713	5	3	0
2015	23	2.363	0.686	5.719	3	0	0

^a^ All LC_50_s expressed as μg of insecticide/vial. ^b^ The highest upper fiducial limit (reported by Snodgrass as confidence limit) is 4.16 μg/vial [20]. ^c^ The upper fiducial limit measured for the Crossett control population in 2010 (Table 1) was 3.564. ^d^ The upper level fiducial limit measured for the Crossett control population in 2014 (Table 1) was 13.895 μg/vial.

**Table 7:** Linear regressions between estimated use (kg ai/ha harvested cropland) of organophosphate, pyrethroid, neonicotinoid, and sulfoxaflor insecticides across all counties/parishes in Arkansas, Louisiana, and Mississippi, 2008–2015.

Dependent Variable	Independent Variable	n	r^2^	Intercept ± SE	Slope ± SE	Model F-Ratio	*p* > F
Organophosphate	Pyrethroid	1087	0.0749	0.0034 ± 0.00044	0.204 ± 0.0218	87.9435	<0.0001
Organophosphate	Neonicotinoid	1024	0.0589	0.0034 ± 0.00049	0.264 ± 0.0330	63.9525	<0.0001
Organophosphate	Sulfoxaflor	316	0.0852	0.0021 ± 0.00056	1.363 ± 0.2579	3.8100	<0.0001
Pyrethroid	Organophosphate	1087	0.0749	0.0090 ± 0.00054	0.367 ± 0.0006	87.9435	<0.0001
Pyrethroid	Neonicotinoid	1415	0.6711	0.00009 ± 0.00036	1.219 ± 0.0277	2885.7100	<0.0001
Pyrethroid	Sulfoxaflor	384	0.2810	0.0102 ± 0.00095	5.627 ± 0.4665	149.3000	<0.0001
Neonicotinoid	Organophosphate	1024	0.0589	0.0073 ± 0.00040	0.223 ± 0.0279	63.9525	<0.0001
Neonicotinoid	Pyrethroid	1415	0.6711	0.0020 ± 0.00019	0.550 ± 0.0102	2885.7000	<0.0001
Neonicotinoid	Sulfoxaflor	384	0.2959	0.0078 ± 0.00057	3.488 ± 0.2753	160.5370	<0.0001
Sulfoxaflor	Organophosphate	316	0.0852	0.00015 ± 0.00010	0.050 ± 0.0001	149.3039	<0.0001
Sulfoxaflor	Pyrethroid	384	0.2810	0.00078 ± 0.00012	0.062 ± 0.0116	29.2378	<0.0001
Sulfoxaflor	Neonicotinoid	384	0.2959	0.00005 ± 0.00011	0.085 ± 0.0067	160.5371	<0.0001

SE: Standard Error.

**Table 8:** Relationships between measured LC_50_s and estimated insecticide use (kg ai/ha harvested cropland) across all individual county-date observations.

Dependent Variable (LC_50_)	Independent Variable (kg ai/ha or LC_50_)	n	r^2^	Intercept ± SE	Slope ± SE	Model F-Ratio	*p* > F
Acephate LC_50_	kg Organophosphates	223	0.0029	11.994 ± 0.7083	61.285 ± 76.0719	0.6490	0.4213
Acephate LC_50_	kg Pyrethroids	223	0.0072	14.093 ± 1.5066	−108.460 ± 85.4267	1.6120	0.2056
Acephate LC_50_	kg Neonicotinoids	223	0.0093	10.688 ± 1.2731	131.968 ± 91.596	2.0758	0.1511
Acephate LC_50_	kg Sulfoxaflor	88	0.0008	12.274 ± 1.6529	−311.993 ± 1198.881	0.0627	0.7953
Acephate LC_50_	Permethrin LC_50_	40	0.1554	6.679 ± 2.8651	1.149 ± 0.4038	8.0958	0.0067
Acephate LC_50_	Imidacloprid LC_50_	137	0.0061	14.336 ± 1.6399	0.544 ± 0.5934	0.8394	0.3613
Acephate LC_50_	Thiamethoxam LC_50_	175	0.0024	12.674 ± 1.1081	0.371 ± 0.5711	0.4206	0.5175
Acephate LC_50_	Sulfoxaflor LC_50_	20	0.2984	−21.881 ± 11.3471	4.919 ± 1.7775	7.6586	0.0127
Permethrin LC_50_	kg Organophosphates	43	0.0583	6.551 ± 1.5400	−896.694 ± 562.7802	2.5387	0.1188
Permethrin LC_50_	kg Pyrethroids	43	0.0641	−4.791 ± 6.2303	686.445 ± 409.7232	2.8699	0.1015
Permethrin LC_50_	kg Neonicotinoids	43	0.0804	−0.216 ± 3.2554	555.668 ± 293.0423	3.5827	0.0655
Permethrin LC_50_	kg Sulfoxaflor	43	0.0450	3.837 ± 1.7699	1411.550 ± 1015.535	1.9370	0.1720
Permethrin LC_50_	Acephate LC_50_	40	0.0001	5.484 ± 2.0152	0.014 ± 0.1303	0.11190	0.9138
Permethrin LC_50_	Imidacloprid LC_50_	42	0.0172	6.659 ± 2.0432	−0.464 ± 0.5543	0.2008	0.4075
Permethrin LC_50_	Thiamethoxam LC_50_	43	0.0068	5.704 ± 1.5103	0.181 ± 0.3392	0.2892	0.5968
Permethrin LC_50_	Sulfoxaflor LC_50_	16	0.1530	0.091 ± 1.1104	0.269 ± 0.1689	2.5348	0.1337
Imidacloprid LC_50_	kg Organophosphates	146	0.0043	2.394 ± 0.2148	−63.165 ± 26.9533	0.6335	0.4274
Imidacloprid LC_50_	kg Pyrethroids	146	0.0254	3.466 ± 0.5343	58.548 ± 30.2102	3.7560	0.0546
Imidacloprid LC_50_	kg Neonicotinoids	146	0.0367	3.272 ± 0.3776	−63.165 ± 26.9533	5.4920	0.0205
Imidacloprid LC_50_	kg Sulfoxaflor	146	0.0117	2.388 ± 0.1938	257.6213 ± 26.9533	1.7098	0.5378
Imidacloprid LC_50_	Acephate LC_50_	113	0.0034	2.682 ± 0.3083	−0.012 ± 0.0209	0.3820	0.5387
Imidacloprid LC_50_	Permethrin LC_50_	48	0.1608	4.403 ± 0.6379	0.2908 ± 0.9079	8.8169	0.0047
Imidacloprid LC_50_	Thiamethoxam LC_50_	136	0.1794	1.923 ± 0.2061	0.367 ± 0.0668	30.1680	<0.0001
Imidacloprid LC_50_	Sulfoxaflor LC_50_	24	0.1682	−1.113 ± 2.6923	0.902 ± 0.4278	4.4494	0.0465
Thiamethoxam LC_50_	kg Organophosphates	202	0.0111	2.224 ± 0.2193	−45.494 ± 30.2289	2.2650	0.1339
Thiamethoxam LC_50_	kg Pyrethroids	202	0.0027	2.469 ± 0.5878	−23.960 ± 32.0533	0.5588	0.4556
Thiamethoxam LC_50_	kg Neonicotinoids	202	0.0025	2.3704 ± 0.4840	−24.995 ± 35.1742	0.5050	0.4782
Thiamethoxam LC_50_	kg Sulfoxaflor	98	0.0000	2.412 ± 0.4393	−26.337 ± 328.0763	0.0065	0.9361
Thiamethoxam LC_50_	Acephate LC_50_	165	0.0032	2.271 ± 0.311	−0.017 ± 0.0241	0.5523	0.4709
Thiamethoxam LC_50_	Imidacloprid LC_50_	147	0.1888	0.398 ± 0.3887	0.761 ± 0.1310	33.7535	<0.0001
Thiamethoxam LC_50_	Permethrin LC_50_	53	0.0001	2.995 ± 0.9224	−0.009 ± 0.1347	0.0051	0.9434
Thiamethoxam LC_50_	Sulfoxaflor LC_50_	23	0.0513	0.7905 ± 1.3932	0.234 ± 0.2199	1.1375	0.2983
Sulfoxaflor LC_50_	kg Organophosphates	21	0.0000	8.990 ± 3.4477	15.800 ± 822.6777	0.0004	0.9849
Sulfoxaflor LC_50_	kg Pyrethroids	21	0.579	−0.307 ± 8.8971	682.563 ± 631.3135	0.0006	0.2931
Sulfoxaflor LC_50_	kg Neonicotinoids	21	0.0483	1.961 ± 6.8501	1574.578 ± 1450.5350	0.9660	0.3380
Sulfoxaflor LC_50_	kg Sulfoxaflor	21	0.0583	3.602 ± 5.9205	4895.250 ± 4980.7199	1.1783	0.2913
Sulfoxaflor LC_50_	Acephate LC_50_	19	0.0067	8.386 ± 2.9777	0.063 ± 0.1864	0.1149	0.7388
Sulfoxaflor LC_50_	Imidacloprid LC_50_	20	0.685	4.214 ± 4.6250	1.203 ± 1.0453	1.3241	0.2649
Sulfoxaflor LC_50_	Thiamethoxam LC_50_	20	0.0057	7.791 ± 4.1424	0.961 ± 2.9999	0.1027	0.7523
Sulfoxaflor LC_50_	Permethrin LC_50_	19	0.0439	0.339 ± 10.274	633.038 ± 716.3655	0.7809	0.3892

**Table 9:** Significant predictors of annual average LC_50_s when LC_50_s and estimated insecticide use were averaged for individual counties and parishes across years (2008–2015).

Dependent Variable	Independent Variable	n	r^2^	Intercept ± SE	Slope ± SE	Model F-Ratio	*p* > F
Permethrin LC_50_	kg Pyrethroids	20	0.5291	−6.497 ± 2.5268	736.429 ± 163.7621	20.2250	0.0003
Permethrin LC_50_	kg Sulfoxaflor	20	0.6332	1.592 ± 0.8345	1981.238 ± 355.3791	31.0886	<0.0001
Imidacloprid LC_50_	kg Pyrethroids	86	0.0599	3.654 ± 0.5580	−70.548 ± 30.2990	19.4873	0.0223
Imidacloprid LC_50_	Thiamethoxam LC_50_	81	0.3938	1.7333 ± 0.2033	0.386 ± 0.0538	51.3111	<0.0001
Thiamethoxam LC_50_	Imidacloprid LC_50_	82	0.4195	−0.445 ± 0.4211	1.013 ± 0.1333	57.8152	<0.0001

**Table 10:** Overview of tarnished plant bug thresholds and recommended insecticides by the Mississippi Cooperative Extension Service for 1983, 1993, 2002, and 2013.

**1983 Recommendations**		
**Plant Bug Thresholds**	**Organophosphates (lb ai/acre)**	
**PreBloom**	acephate (0.33)	
1 bug/3 row feet (drop cloth)	azinphosmethyl (0.16–0.25)	
15 bugs/100 plant terminals (visual)	chloropyrifos (0.25–0.5)	
30 bugs/100 sweeps (sweep net)	dicrotophos (0.1–0.2)	
**PostBloom**	malathion (0.7–1.25)	
2 bugs/3 row feet (drop cloth	methyl parathion (0.25–0.5)	
20 bugs/100 terminals (visual)	monocrotophos (0.1–0.2)	
60 bugs/100 sweeps (sweep net)	sulprofos (0.25–0.33)	
	trichlorfon (0.25–0.5)	
	**Carbamates (lb ai/acre)**	
	carbaryl (0.7–1.4)	
**1993 Recommendations**		
**Plant Bug Thresholds**	**Organophosphates (lb ai/acre)**	
**PreBloom: 1st two weeks squaring**	acephate (0.25–0.33)	
1 bug/6 row feet (drop cloth)	azinphosmethyl (0.16–0.25)	
5 bugs/100 plant terminals (visual)	chloropyrifos (0.20–0.50)	
7.5 bugs/100 sweeps (sweep net)	dicrotophos (0.10–0.20)	
**PreBloom: after 3rd week squaring**	dimethoate (0.10–0.20)	
1 bugs/3 row feet (drop cloth	malathion (0.70–1.25)	
10 bugs/100 terminals (visual)	encapsulated methyl parathion (0.25–0.5)	
15 bugs/100 sweeps (sweep net)	methyl parathion (0.25–0.5)	
**PostBloom**	sulprofos (0.25–0.33)	
2 bugs/3 row feet (drop cloth	profenofos (0.25–0.33)	
15 bugs/100 terminals (visual)		
30 bugs/100 sweeps (sweep net)	**Carbamates (lb ai/acre)**	
	oxyamyl (0.02–0.25)	
**2003 Recommendations**		
**Plant Bug Thresholds**	**Organophosphates (lb ai/acre)**	
**PreBloom: 1st two weeks squaring**	acephate (0.25–0.33)	
1 bug/6 row feet (drop cloth)	dicrotophos (0.10–0.20)	
5 bugs/100 plant terminals (visual)	malathion ULV (0.92–1.22)	
8 bugs/100 sweeps (sweep net)	methamidophos (0.33–0.50)	
**PreBloom: after 3rd week squaring**		
2 bugs/3 row feet (drop cloth	**Neonicotinoids (lb ai/acre)**	
10 bugs/100 terminals (visual)	imidacloprid (0.047)	
15 bugs/100 sweeps (sweep net)	thiamethoxam (0.02)	
**PostBloom**		
3 bugs/3 row feet (drop cloth	**Carbamates (lb ai/acre)**	
15 bugs/100 terminals (visual)	oxamyl (0.25–0.33)	
30 bugs/100 sweeps (sweep net)		
**2013 Recommendations**		
**Plant Bug Thresholds**	**Organophosphates (lb ai/acre)**	**Carbamates (lb ai/acre)**
**PreBloom: 1st two weeks squaring**	acephate (0.5–1.0)	oxamyl (0.33–0.50)
1 bug/6 row feet (drop cloth)	dicrotophos (0.25–0.50)	
5 bugs/100 plant terminals (visual)	dimethoate (0.25–0.50)	**Pyridinecarboxamides (lb ai/acre)**
8 bugs/100 sweeps (sweep net)	malathion (0.92–1.22)	flonicamid (0.054–0.089)
**3rd week squaring through bloom**		
3 bugs/3 row feet (drop cloth)	**Neonicotinoids (lb ai/acre)**	**Insect Growth Regulator (lb ai/acre)**
10 bugs/100 terminals (visual)	acetamiprid (0.05)	novaluron (0.04–0.06)
15 bugs/100 sweeps (sweep net)	clothianidin (0.02–0.10)	
	imidacloprid (0.047–0.062)	**Sulfoximines (lb ai/acre)**
		sulfoxaflor (0.0375–0.0625)

**Table 11:** Significant correlations observed between measured LC_50_s, estimated insecticide use per acre of harvested cropland, and cooperative extension service estimates of cotton insect losses and control costs for 2008–2015.

Variable 1	Variable 2	Number	Correlation Coefficient (r)	*p* > r
Total number foliar applications	Number of applications for plant bugs	24	0.7120	<0.0001
Cost of all foliar application	Number of applications for plant bugs	24	0.6046	0.0018
Cost of all foliar application	Total number foliar applications	24	0.7209	0.0283
Permethrin LC_50_	Thiamethoxam LC_50_	5	0.8998	0.0375
Acephate LC_50_	Yield	20	−0.4480	0.0476
kg Organophosphates	Yield	24	−0.4938	0.0142
